# Mechanistic Insights Into Instability From Single Void Dynamics at the Lithium Metal‐Solid Electrolyte Interface

**DOI:** 10.1002/advs.78028

**Published:** 2026-09-30

**Authors:** Sourim Banerjee, Bairav S. Vishnugopi, Guang Lin, Partha P. Mukherjee

**Affiliations:** ^1^ School of Mechanical Engineering Purdue University West Lafayette Indiana USA; ^2^ Department of Mathematics Purdue University West Lafayette Indiana USA

**Keywords:** lithium metal anode, single void evolution, solid‐state batteries, surface diffusion

## Abstract

Among emerging energy storage technologies, lithium (Li) metal anode‐based solid‐state batteries (SSBs) have drawn considerable attention, as they promise greater energy density alongside improved safety relative to traditional Li‐ion cells. Despite this promise, the performance of such systems remains constrained by intrinsic obstacles tied to the nucleation and growth of voids at the Li‐solid electrolyte (SE) interface as stripping proceeds. Void morphology and contact distribution jointly dictate the coupled reaction‐diffusion behavior at the interface, thereby affecting the interfacial resistance. In the present study, we track the detailed evolution of a single void as Li stripping progresses, characterizing how its shape, size, and overall morphology evolve under the action of the distinct modes of surface diffusion, i.e., terrace, step, and interlayer diffusion. The depth and size attained by the void are strongly governed by coupling between reaction kinetics and the surface diffusion modes, which together dictate the dominant diffusion pathways. In addition, the role of operating temperature in mitigating void growth under high‐reaction‐rate conditions is examined. Extending the single‐void analysis to the collective voids regime, we resolve the progression toward a multi‐clustered morphology and determine the critical void fraction value the onset of percolation, beyond which dispersed voids coalesce into a connected network. Understanding these interactions between voids helps identify the mechanisms that control the final interfacial structure and provides guidance for designing stable, low‐resistance interfaces in SSBs.

## Graphical abstract

Void morphology at the lithium metal‐solid electrolyte contact governs electrochemical contact stability in all‐solid‐state batteries. This work resolves the competing roles of electrochemical reaction, bulk vacancy diffusion, and distinct surface diffusion pathways in dictating the size and morphology evolution of a single void and its transition to a percolated void network.

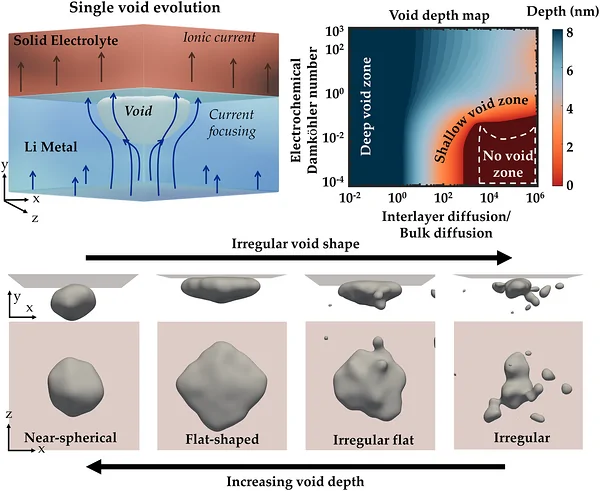

## 1 Introduction

The widespread use of portable electronics and electric vehicles has driven the rapid adoption of rechargeable Li‐ion batteries in today's technological world [1]. To meet the increasing energy demands, solid‐state batteries (SSBs) employing lithium (Li) metal anodes have emerged as a promising route for next‐generation energy storage [2]. Li metal is regarded as a leading anode candidate, combining a specific capacity as high as 3860 mAh g^−^^1^ with a low mass density (0.534 g cm^−^^3^), and a strong reducing redox potential (−3.04 V vs. standard hydrogen electrode), which together position it as one of the most compelling options for commercialization in next‐generation cells [3, 4, 5, 6]. These attributes have renewed strong interest in Li metal for advanced rechargeable systems and for high‐energy chemistries beyond current Li‐ion technology, including Li–S and Li–air batteries [7, 8, 9]. In parallel, inorganic solid electrolytes (SEs) offer mechanical robustness, selective Li‐ion transport, nonflammability, suppression of parasitic electrode cross‐talk, and the potential for improved thermal and rate performance [10, 11, 12]. Consequently, SSBs incorporating Li metal anodes are widely regarded as a strong contender for next‐generation batteries with enhanced energy density and improved safety. Nevertheless, the practical realization of SSBs remains impeded by intrinsic limitations originating from the interplay of electrochemical reactions, transport phenomena, and mechanical effects at the Li‐SE interface [13].

Interfacial stability at the Li–SE boundary critically governs the performance and reliability of SSBs. The solid–solid nature of this interface promotes spatially heterogeneous Li deposition, in which localized current focusing and transport limitations trigger filament growth that penetrates the electrolyte via defects, grain boundaries, pores, and microcracks, ultimately leading to internal short‐circuiting [14, 15, 16, 17]. The deposition behavior is strongly influenced by interfacial morphology and electrolyte microstructure, where local inhomogeneities and imperfect contact amplify reaction heterogeneity and current localization [18, 19, 20, 21]. The inherent solid‐solid non‐conformal contact is responsible for heterogeneous current distribution and therefore non‐homogeneous deposition as well as electro‐dissolution. Furthermore, interfacial evolution during cycling, intensifies current focusing and destabilizes the buried interface [9, 22, 23]. These coupled electrochemical, transport, and mechanical effects collectively promote contact loss, particularly during Li stripping [24, 25, 26]. Consequently, interfacial void formation during stripping emerges as a critical phenomenon governing contact loss and subsequent electro‐chemo‐mechanical instability at the Li–SE interface [27, 28].

Experimental studies have demonstrated void formation at the Li–SE interface as a dominant degradation pathway during electrochemical stripping. Early work on Li‐Li_7_La_3_Zr_2_O_12_ (LLZO) SE showed that the stripping response is strongly pressure‐dependent and that polarization rises sharply when the stripping rate exceeds the ability of Li to replenish the interface, indicating that void formation and contact loss drive the elevated resistance [29, 30]. Three‐electrode studies on sulfide electrolytes refined this picture by decoupling stripping from plating and identifying a critical stripping current above which voids accumulate, reduce active contact area, intensify local current density, and promote dendrite formation during subsequent plating [31, 32]. Operando synchrotron X‐ray tomography then provided direct evidence that stripping‐induced contact loss, rather than interphase growth alone, is the primary cause of current constriction and cell failure [33]. More recent operando and in‐situ techniques have further clarified the dynamics of void evolution. Operando dilatometry showed that pore formation can begin immediately upon stripping, even before conventional impedance measurements clearly detect degradation, and suggested that pores initially expand laterally before deepening [34]. In‐situ optical and electron microscopy on the Li–SE interface demonstrated that void density, morphology, and electrochemically active area depend strongly on current density, applied load, and Li microstructure [35]. Studies on thin, in situ‐formed Li anodes further showed that unstable stripping is not fully reversed by replating and that thermal treatment can partially restore the de‐wetted solid–solid interface, highlighting the persistence of interfacial damage [36]. Pressure‐ and temperature‐dependent studies similarly showed that creep‐assisted deformation can mitigate voiding, but pressure alone is often insufficient to eliminate irrecoverable voids under high‐capacity stripping [37]. Complementing these observations, operando Galvanostatic Electrochemical Impedance Spectrocopy (GEIS)‐based diagnostics have enabled earlier detection of void nucleation through frequency‐dependent impedance signatures. At the same time, multiscale electrochemical and microscopic studies have mapped how current density and areal capacity control void nucleation density, growth mode, and contact‐loss progression [38, 39]. Collectively, these experiments show that stripping‐induced void evolution is governed by current density, stack pressure, temperature, interfacial chemistry, and microstructural heterogeneity, all of which regulate the loss and possible recovery of electrochemically active contact [40].

This experimental understanding has motivated extensive modeling efforts to identify the governing mechanisms of void nucleation, growth, collapse, and coalescence. Foundational electro‐chemo‐mechanical analyses of electrochemically active solid–solid interfaces have argued that vacancy accumulation under an anodic load imposes an intrinsic morphological instability and that stable operation is constrained by a critical stripping condition linked to vacancy transport in the Li anode [30]. Subsequent mechanics‐based studies showed that conventional Butler–Volmer kinetics alone cannot capture experimentally observed void initiation, and that creep, dislocation effects, and pressure‐dependent interfacial kinetics must be included to describe void growth [41, 42]. Phase‐field electro‐chemo‐mechanical models have since expanded this framework by coupling vacancy nucleation and annihilation, bulk and surface Li transport, visco‐plastic creep, interfacial contact evolution, and electrolyte current redistribution, thereby reproducing experimentally observed pressure and current effects while also predicting local current hot spots that connect stripping‐side voiding to plating‐side dendrite initiation [43, 44]. Other studies have shown that interfacial stability is not governed by bulk diffusion alone. Multiscale DFT‐KMC‐FEM analyses demonstrated that interface chemistry and lithiophilicity strongly influence steady‐state contact loss and the stack pressure required for stable stripping. In this context, atomistic and KMC studies revealed that lithiophilic interfaces drive vacancies into the bulk, whereas lithiophobic interfaces trap vacancies near the interface and promote void formation [45, 46, 47]. Additional modeling has highlighted the roles of creep‐enhanced vacancy transport, surface diffusion, temperature, and local heterogeneities such as grain boundaries in determining void morphology, contact evolution, and stripping hot spots [28, 48, 49, 50]. At the same time, some theoretical and experimental studies have challenged the assumption that all preexisting voids necessarily grow under stripping, suggesting instead that isolated voids may collapse under creep or shrink by Li migration, and that severe degradation may require impurity‐assisted initiation or void coalescence rather than monotonic growth of a single defect [40, 42]. However, these models still fall short of fully capturing the morphological and geometric evolution of voids at smaller length scales, where localized transport and interfacial processes govern void morphology, shape, and size, which is critical to understanding interfacial instability. Therefore, a more detailed and comprehensive understanding is essential.

In this context, examining the evolution of a focused single void offers valuable insights for revealing how void geometry, morphology, and shape emerge from the coupled effects of surface diffusion, vacancy diffusion, electrochemical reaction, and temperature. Building on this perspective, we develop a mesoscale Kinetic Monte Carlo (KMC) framework that captures the competing mechanisms governing interfacial evolution during stripping, including electro‐dissolution at active contact sites, vacancy transport within the Li metal, and surface‐mediated relaxation of the evolving void boundary. As these processes occur, the void continuously changes its size, shape, and local curvature, thereby altering interfacial contact, current distribution, and morphological stability. Using this framework, we systematically analyze the effects of surface diffusion pathways, operating temperature, and overpotential on single‐void evolution. We further examine how an initially isolated void progressively interacts with neighboring depleted regions to form a percolated void network, and how this percolation behavior evolves as stripping progresses. Such an approach provides a mechanistic bridge between isolated interfacial instability and the emergence of collective contact loss that ultimately governs degradation in solid‐state batteries.

## 2 Methodology

During metal electro‐dissolution, a Li atom in contact with the SE undergoes electrochemical oxidation. It produces a Li^+^ ion inside a SE interstitial site and an electron (e^−^) in the anode, and it leaves behind a vacancy site (V_Li_) at the interface. This electrochemical reaction can be written using the Kröger–Vink notation [51],

(1) $$Li_{Li}^{\times }\to Li_{SE}^{+}+e^{-}+V_{Li}^{\times }$$

The intrinsically solid–solid character of the interface makes the dissolution reaction heterogeneous across the interface, thereby expediting vacancy accumulation, concentration gradients, and nonuniform current distribution. As stripping progresses, the steady depletion of Li atoms at the contact seeds new voids along the solid–solid boundary. As preexisting vacancies migrate into the bulk, additional vacancies are generated concurrently at neighboring sites that remain electrochemically active. The resulting defect population and spatial configuration are modulated by available transport pathways, dominant atomic diffusion pathways along the surface, and the spatially nonuniform arrangement of metal atoms. The evolving Li–SE contact, together with the stochastic nature of these processes, controls the nucleation and subsequent growth of interfacial voids.

In this work, a three‐dimensional KMC model is developed to examine the progression of a single hemispherical void, tracking its structural evolution under the influence of surface diffusion and reaction kinetics. LLZO serves as the representative SE in this work, and the competing mechanisms incorporated in this model near the Li–SE interface, shown in Figure 1a,b, are as follows,

- Mechanism 1: Intrinsic bulk migration of Li vacancies from the Li–SE interface toward the bulk electrode.
- Mechanism 2: Surface diffusion of Li atoms across the uppermost layer of the Li metal surface.
- Mechanism 3: Electro‐dissolution at the active contact sites, described through Butler–Volmer kinetics [52].
(2) $$J=i_{0}\;\left(\exp\left(\frac{\alpha F}{RT}\eta \right)-\exp\left(-\frac{\beta F}{RT}\eta \right)\right)$$
- Mechanism 4: Transport of Li^+^ ions from the interface into the bulk SE.

where α and β are the charge transfer coefficients, R is the gas constant, T is the operating temperature, F is Faraday's constant, and η is the local overpotential driving the electrochemical reactions. The value of the η is fixed at 0.8 V [53], which can be considered on the higher side of the range, unless specified. *i*_0_ is the exchange current density for the anode stripping reaction and is independent of operating temperature, and *J* is the electrode current density.

**FIGURE 1:**
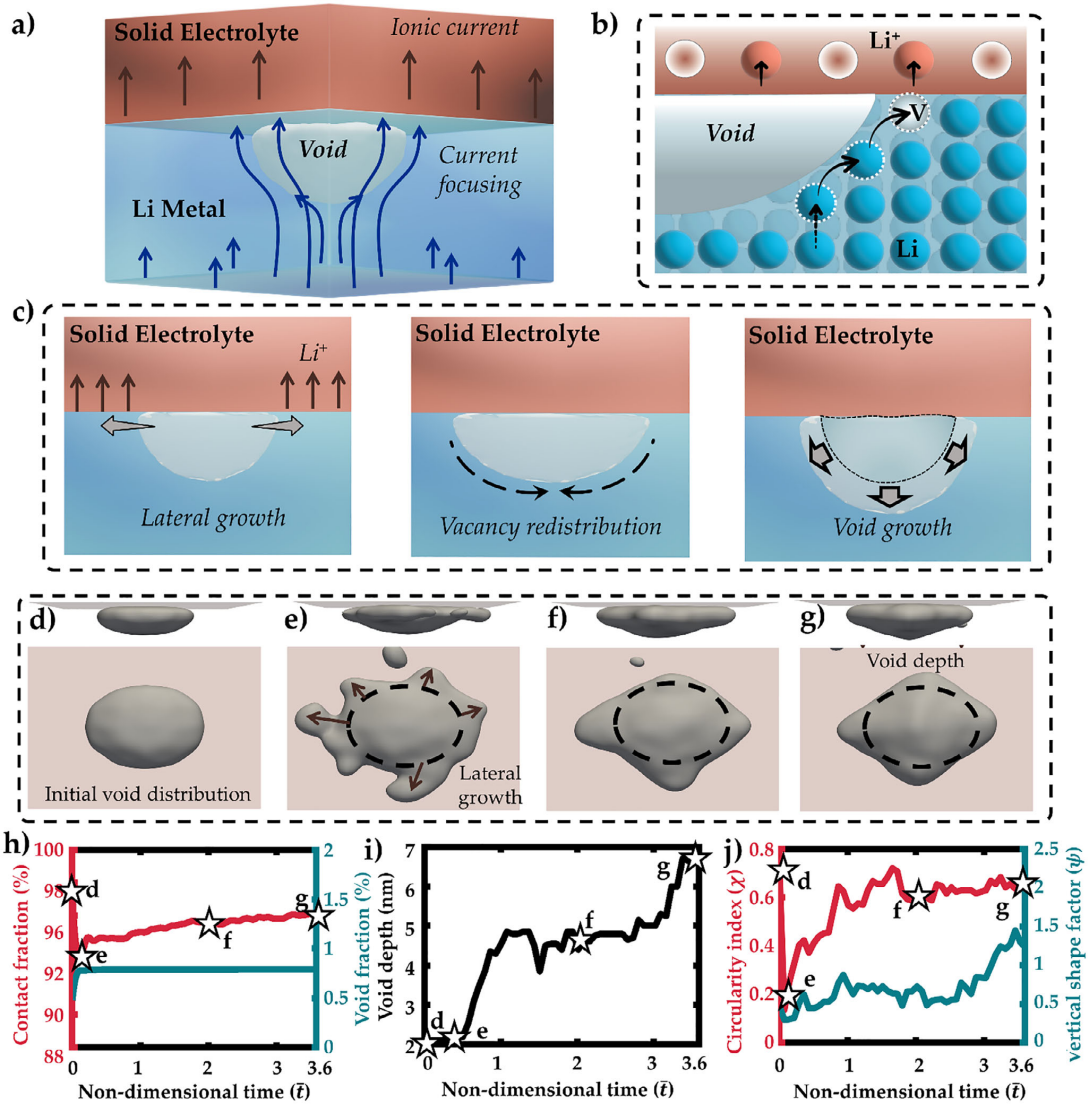
(a) Cross‐sectional schematic of the Li–SE interface with a single void embedded in the Li metal anode, illustrating current focusing near the void edge. (b) Schematic of an atomic‐scale view of surface diffusion of Li atoms and electrochemical oxidation reactions around the void. (c) A mechanistic schematic of the single‐void dynamical growth that shows lateral void growth followed by vacancy redistribution. (d–g) Snapshots of the side‐view and cross‐sectional view of the dynamical evolution of single‐void morphology. Dynamical evolution of the (h) contact fraction, void fraction, (i) void depth, (j) circularity index, and vertical shape factor due to the single‐void evolution.

Surface diffusion is central to the developing void morphology, especially over the corrugated anode surface, by shuttling Li atoms along the interface and therefore moderating local curvature gradients, dictating the geometry and spatial trajectory of the void throughout stripping. In this work, three modes of surface diffusion are incorporated, influenced by the terrace‐step‐kink (TSK) theoretical framework [50, 54], which are as follows:

1. Terrace diffusion: an adatom hops across a flat crystalline plane, or terrace.
2. Step diffusion: an adatom moves along a step edge on the crystal surface.
3. Interlayer diffusion: an atom transfers between adjacent atomic layers, generally by traversing a step edge. This diffusion may proceed in a downward (descending) or upward (ascending) direction.

For a BCC Li crystal, the terrace and step diffusion occur along the family of <100> directions, and interlayer diffusion occurs along the family of <111> directions. The three surface diffusion modes are distinguished through the first nearest neighbor (1NN) and second nearest neighbor (2NN) atomic coordination numbers of the initial and destination sites of each attempted move, following the classification scheme of our earlier work [50]. A surface diffusion move is assigned to terrace diffusion when there is no net change in the 1NN and 2NN atomic coordination and the neighboring atoms remain on the same side of both the initial and destination sites. The event is associated with step diffusion when the destination site acquires one additional 2NN atomic neighbor and more than one 1NN atomic neighbor, and with interlayer diffusion when a descending move into an adjacent layer maintains or increases the combined 1NN and 2NN atomic coordination. Bulk vacancy migration proceeds independently of coordination, so the surface diffusion pathways activate only along the void boundary and the free surface.

As oxidation proceeds, vacancies are steadily transported from the interfacial region towards the anode bulk. At every lattice site along the electrode–electrolyte boundary, the reaction rate (*k_R_*) is prescribed as:

(3) $$k_{R}=\left\{\begin{array}{c} 0\\ k_{0} \end{array}\right.\;\begin{array}{c} \mathrm{if}\;\mathrm{vacancy}\\ \mathrm{if}\;\mathrm{Li}\;\mathrm{atom} \end{array}$$

A site on the electrode's top surface that is not occupied by a Li atom is assigned zero reaction rate (*k_R_* =  0). In contrast, when a Li atom resides at that site, the reaction rate takes the value of the intrinsic reaction rate, *k_R_* = *k*_0_ , expressed as follows:

(4) $$k_{0}=\frac{Ja^{2}}{F}\;N_{a}$$

Here *a* is the lattice constant for Li, and *N_a_* is the Avogadro constant. In this model, the presence of a void at the interface significantly distorts the local potential distribution within the electrolyte, concentrating the current flux in the immediate vicinity of the void boundary and making these regions more prone to electrochemical dissolution. This current focusing enhancement decays spatially following a 1/*r* ^3^ dependence for a non‐conducting hemisphere, where r is the distance from the void center. Based on this decay, the current density returns to within ∼6% [55] of the far‐field value at five lattice spacings beyond the void edge for the initial void radius, providing a physically motivated threshold. Accordingly, the dissolution reaction rate *k_R_* =  *k*_0_ is applied to Li atoms within five lattice spacings (∼1.75 nm) of the nearest void boundary at the Li/SE interface; atoms beyond this zone are assigned *k_R_* =  0. According to the transition state theory [56], Li atom bulk diffusion follows Arrhenius behavior, and the corresponding rate (*k_D_*) is given by:

(5) $$k_{D}=\nu \exp\left(-\frac{E_{a,D}}{k_{b}T}\right)$$

Here, ν denotes the hopping frequency [57] (ranging between 10^12^ to10^13^ *s* ^−1^), *k_b_* is the Boltzmann constant, and *E*_*a*,*D*_ is the barrier for a Li atom to hop between neighboring lattice sites in the Li metal, set to 0.41 eV in this study [54, 58]. Vacancy migration is taken to occur in the opposite direction to Li atom hopping. The surface diffusion rate (*k_SD_*) of atoms on the Li metal surface follows an analogous form:

(6) $$k_{SD}=\nu \exp\left(-\frac{E_{a,SD}}{k_{b}T}\right)$$

The activation barriers for the three surface diffusion mechanisms are adopted from the literature, namely 0.3 eV for terrace diffusion (*E*_*a*,*T*_), 0.15 eV for step diffusion (*E*_*a*,*S*_) and 0.5 eV for interlayer diffusion (*E*_*a*,*I*_) [54]. Within the SE, the ionic diffusion rate (*k_IT_*), which captures ion hopping between adjacent interstitial sites, takes a similar form:

(7) $$k_{IT}=\nu \;\exp\left(-\frac{E_{a,IT}}{k_{b}T}\right)$$

The activation energy governing ion migration through the SE is set to 0.35 eV [59, 60].

To account for temperature dependence, the Arrhenius expressions in Equations (5)–(7) are generalized so that the temperature‐sensitive quantities, i.e., ion diffusion rate in the SE (*k_IT_*), Li bulk diffusion rate (*k_D_*), surface diffusion rate (*k_SD_*), evolve with temperature according to:

(8) $$X_{T}=X\exp\left(-\frac{E_{a,X}}{k_{b}}\left(\frac{1}{T}-\frac{1}{T_{0}}\right)\right)$$

In this relation, *X_T_* is the value of a given temperature‐dependent parameter at an arbitrary temperature T, while X is its value at the reference temperature *T*_0_, set to 27°C here.

The computational domain is modeled as a 50 × 50 × 50 cubic lattice, equivalent to a cuboidal cell measuring 17.5 nm per side. Along the x and z directions transverse to the electrode depth, periodic boundary conditions are applied. The KMC timestep is evaluated from:

(9) $$\Delta t=-\frac{\ln\left(r\right)}{K}$$

where r is a uniformly distributed random number, and K is the cumulative sum of all reaction and transport rates over a given step (Supporting Information Equation S5). To track the extent of Li stripping, a dimensionless time $\bar{t}$ is defined as:

(10) $$\bar{t}=\left(t+\Delta t\right)\;\times k_{D_{0}}$$

Here, $k_{D_{0}}$ is the room‐temperature bulk diffusion rate of Li metal, obtained from the Li bulk diffusivity, *D_Li_* =  2*10^−14^ m^2^/s through:

(11) $$k_{D_{0}}=D_{Li}\;/a^{2}$$

Further details of the KMC scheme are given in the Supporting Information (Figure S1), and the complete set of model parameters is listed in Table S1.

## 3 Results and Discussion

### 3.1 Morphological Development of a Single‐Void During Stripping

The vacancy diffusion flux, surface diffusion along the void surface, and the interfacial reaction dynamics collectively govern the dynamical evolution of an existing hemispherical single void. Existing experimental study by Schall et al. [34]. has inspired us to develop a detailed, three‐step process for individual void growth, shown in Figure 1c. First, the void is initiated at the solid–solid interface as a hemispherical shape, with its circular edge attached to the interface. Second, the void grows laterally along the interface due to high electrochemical stripping around the void edge, and third, due to the redistribution of vacancies from the interface towards the electrode depth. Figure 1d–g shows four snapshots of void evolution from our KMC simulations, which follow the hypothesis mentioned just before. The initial void is initialized to be a hemispherical shape with a circular cross‐section at the Li–SE interface. As shown in Figure 1a, the electronic current is concentrated at the void edges due to charge conservation around the void. Therefore, the edges are more prone to electrochemical stripping. In Figure 1e, the void edges are deformed along the interface because of current focusing and promote lateral growth. With time, vacancy diffusion and surface diffusion kick in, and vacancy redistribution takes place from the interface to the bottom of the void along the void surface, which is presented in Figure 1f. At the end of our simulation, in Figure 1g, the void edges at the interface are retracted relative to Figure 1e, and the void depth increases significantly compared to the initial configuration. So, a single void growth can be explained by two distinct step‐by‐step processes. First, the lateral growth of the void along the interface, and then an increase in the void depth due to the redistribution of the vacancies. The sensitivity to the initial void geometry is examined in Section S3 by varying both the initial radius and aspect ratio. Across these conditions, the final morphological regime remains unchanged.

Changes in contact area, pore volume, and related geometric descriptors are introduced to characterize this evolution of void morphology and geometry. In Figure 1h, the contact area fraction and the void fraction within the anode are tracked as functions of time, where the four instances described earlier are clearly visible. The lateral growth of the void is clearly shown when the contact fraction decreases steeply, and the void fraction also increases significantly. Later, during redistribution, the contact fraction increases as interfacial contact is restored. Still, the void fraction does not change significantly as there are fewer atoms stripped during that time interval. Similarly, in Figure 1i the void depth does not change during lateral growth, but it changes in steps as vacancies redistribute due to surface diffusion. In Figure 1j, there are two geometric descriptors introduced, circularity index (χ) and vertical shape factor (ψ) defined below:

(12) $$\chi =\frac{4\pi A}{P_{c}^{2}}\;$$

(13) $$\psi =\frac{b}{\sqrt{R_{x}R_{z}}}\;$$

where, *b* is the maximum depth of the void in the depth (here, y) direction, *R_x_*, *R_z_* are the maximum distances of the void edge from the geometric center of the void along the x and z directions, respectively. *A* is the cross‐sectional area covered by the void at the Li–SE interface, *P_c_* is the corrected perimeter traced by the void at the interface. The circularity index χ quantifies the in‐plane compactness of the void footprint at the interface. For reference, χ = 1 is associated with a perfect circular cross‐section, and values below 1 indicate deviation from circularity due to elongation or surface roughness. As the void footprint is on a square grid, the corrected perimeter *P_c_* ( = P*π/4, where P is the perimeter measured by counting exposed cell‐edges on the square grid and π/4 corrects for the systematic overestimation relative to a smooth continuous boundary) is used to calculate χ. Since the void footprint spans approximately five lattice spacings in radius, the correction is not fully converged, and the practical maximum χ for a circular footprint on this lattice is approximately 0.72 rather than the theoretical value of 1. Values near the lattice maximum of approximately 0.72 correspond to a compact, near‐circular footprint, and decreasing χ reflects elongation or edge roughening produced by irregular stripping. Later, χ increases again, showing a re‐emergence of a more symmetric cross‐section driven by surface diffusion. The vertical shape factor ψ compares the maximum depth b with the geometric mean lateral radius. ψ ≪ 1 corresponds to a flat void spread along the interface, ψ ≈ 1 to a near‐hemispherical void, and ψ  >  1 to a void elongated into the electrode depth. With continued stripping, ψ remains approximately constant during lateral growth when depth (*b*) is unchanged, and increases significantly during the redistribution regime as the void deepens.

### 3.2 Effect of Surface Diffusion Modes on Single Void Depth and Size

In this section, the final void depth and sizes are characterized at the end of the stripping simulation, and how the surface diffusion modes shape the resulting morphology is examined. Figure 2a–c presents phase maps of void depth against the terrace (*E*_*a*,*T*_) and interlayer diffusion (*E*_*a*,*I*_) activation barriers, evaluated at three step diffusion activation barriers (*E*_*a*,*S*_) of 0.05, 0.2, 0.5 eV. The two distinct regimes in these phase plots are the detached and attached voids. The no‐contour brown regions correspond to the “detached void,” which is not attached to the interface but has been moved into the electrode bulk. In the remaining region, the void is attached to the interface, and therefore, the contact area fraction is less than 1. The void depth is calculated as the maximum distance of the void from the interface. Likewise, the void size is calculated as the maximum width of the void along the depth (here, y) direction, and this definition applies to both attached and detached voids. For the “attached void” case, void size and void depth denote the same value. In Figure 2a–f, we highlight a few examples with extreme values of void depth and size, and the corresponding void morphologies are shown in Figure 2g–p. In Figure 2a, the void depth values are not at their extremes at the boundary values of *E*_*a*,*T*_, *E*_*a*,*I*_  =  0.05, 0.5 eV. However, if the value is changed from *E*_*a*,*T*_  =  0.05 eV, *E*_*a*,*I*_  =  0.05 eV (Figure 2g) to *E*_*a*,*T*_  =  0.05 eV, *E*_*a*,*I*_  =  0.5 eV (Figure 2h), the void becomes more flat‐shaped from a spherical shape. Therefore, a suppressed interlayer diffusion mode with terrace diffusion remaining active promotes a flat void shape. Similarly, in the case where *E*_*a*,*T*_  =  0.5 eV, *E*_*a*,*I*_  =  0.5 eV (Figure 2i), the void increases both in depth and along the interface. That means higher step diffusion boosts void expansion in both directions. Now, the highest depth in Figure 2a occurs at *E*_*a*,*T*_  =  0.2 eV, *E*_*a*,*I*_  =  0.2 eV (Figure 2j), around 4 nm, which is a more rounded shape. From this analysis, it can be concluded that if both. *E*_*a*,*T*_, and *E*_*a*,*I*_ are equal and very low in value, the void shape is very spherical, and as the value increases, the void becomes flatter as the step diffusion becomes more dominant. However, the detached void region is predominantly in the high interlayer diffusion region, and this trend is more visible as the *E*_*a*,*S*_ value increases. This is because, with the increasing dominance of interlayer diffusion, the vacancies are more likely to be transported in the depth direction and therefore more prone to forming a detached void. Now, in Figure 2d, examining the detached void zone, two extreme regions appear. At *E*_*a*,*T*_  =  0.4 eV, *E*_*a*,*I*_  =  0.2 eV (Figure 2k), the void is deformed in the flat region. The deformed shape results from the combined effects of interlayer and step diffusion. At *E*_*a*,*T*_  =  0.4 eV, *E*_*a*,*I*_  =  0.05 eV (Figure 2l), the void is elongated along the depth direction due to the strong influence of interlayer diffusion.

**FIGURE 2:**
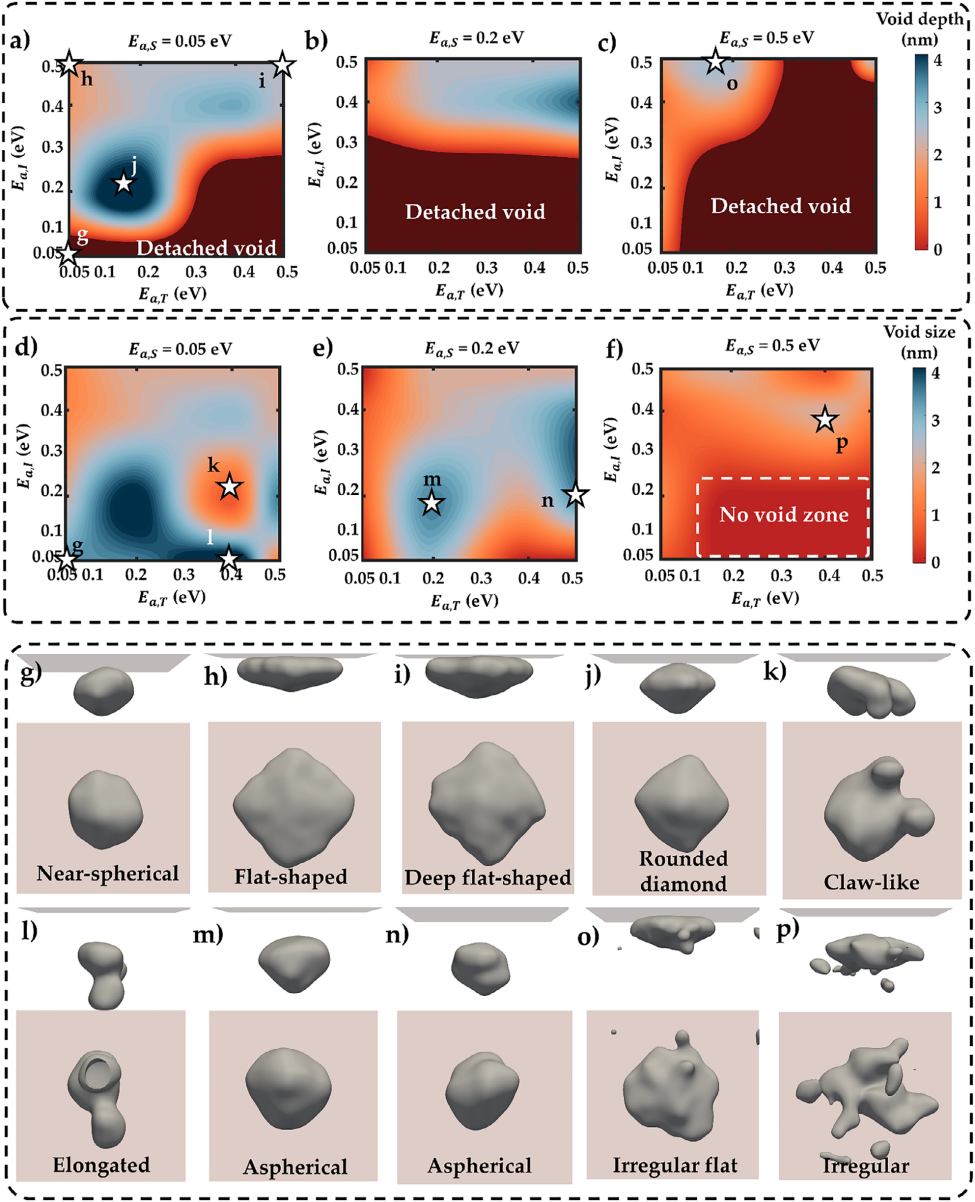
(a–c) Void depth phase maps as a function of *E*_*a*,*T*_ and *E*_*a*,*I*_ for *E*_*a*,*S*_  =  0.05, 0.2, 0.5 eV. (d–f) Void‐size phase maps as a function of *E*_*a*,*T*_ and *E*_*a*,*I*_ for *E*_*a*,*S*_  =  0.05, 0.2, 0.5 eV. (g–p) Distinct shapes and morphologies of the single‐void for different combinations of surface diffusion activation barriers.

In Figure 2b, the detached void region significantly increased as *E*_*a*,*S*_ changes from 0.05 to 0.2 eV. This indirectly indicates that interlayer diffusion becomes more influential than the other surface diffusion modes, and therefore the voids become detached with a lower *E*_*a*,*I*_ value. Focusing more on the detached void regime, there are a couple of examples with a large void size at *E*_*a*,*T*_  =  0.2 eV, *E*_*a*,*I*_  =  0.2 eV (Figure 2m), and *E*_*a*,*T*_  =  0.5 eV, *E*_*a*,*I*_  =  0.2 eV (Figure 2n). Voids are aspherical in both cases, but Figure 2m is more spherical compared to Figure 2n, and this is because the interlayer diffusion is dominant for the latter. In Figure 2c, the detached void regime again extended for the same reason as before, however, at *E*_*a*,*T*_  =  0.2 eV, *E*_*a*,*I*_  =  0.5 eV (Figure 2o), the void is attached to the interface, but the void surface is rough due to reduced step diffusion. Similarly, in Figure 2f, the detached void regime has a peculiar extreme point at *E*_*a*,*T*_  =  0.4 eV, *E*_*a*,*I*_  =  0.4 eV (Figure 2p), where the void is more ramified due to the relaxed step diffusion. In Figure 2f, there is a void size = 0 regime, which indicates that the voids are completely diffused through the Li anode due to the combined dominance of interlayer and bulk diffusion. A global variance‐based sensitivity analysis of the three surface diffusion activation barriers on the final void depth is presented in Section S2. The first‐order, total‐order, and second‐order Sobol indices are reported in Figure S2, and their convergence with sample size is verified in Figure S3.

### 3.3 Temperature vs. Reaction Kinetics Impact on the Single Void Morphology

Figure 3 shows the competing effects of temperature and reaction kinetics that dictate the single‐void morphology. The rate of the electrochemical dissolution reaction is governed by changes in overpotential as described by Butler–Volmer reaction kinetics (Equation 2). Changing the temperature will affect bulk vacancy diffusion and surface diffusion kinetics, as described by Equation 8. In Figure 3a–e, first the *E*_*a*,*I*_ is changed from 0.05 to 0.5 eV keeping *E*_*a*,*T*_  =  0.05 eV, *E*_*a*,*S*_  =  0.5 eV constant and then *E*_*a*,*T*_ is changed from 0.05 to 0.5 eV keeping *E*_*a*,*S*_  =  0.5 eV, *E*_*a*,*I*_  =  0.5 eV constant and the void depth is plotted by changing the temperature from −10°C to 80°C, and the overpotential is varied from 0.05 to 1 V. This phase space spans from sub‐zero operational temperatures to extremely high temperatures. However, in the case of overpotential, the practical range of the same varies till 0.3–0.4 V at maximum, but this study explores till 1 V, which is an extreme case.

**FIGURE 3:**
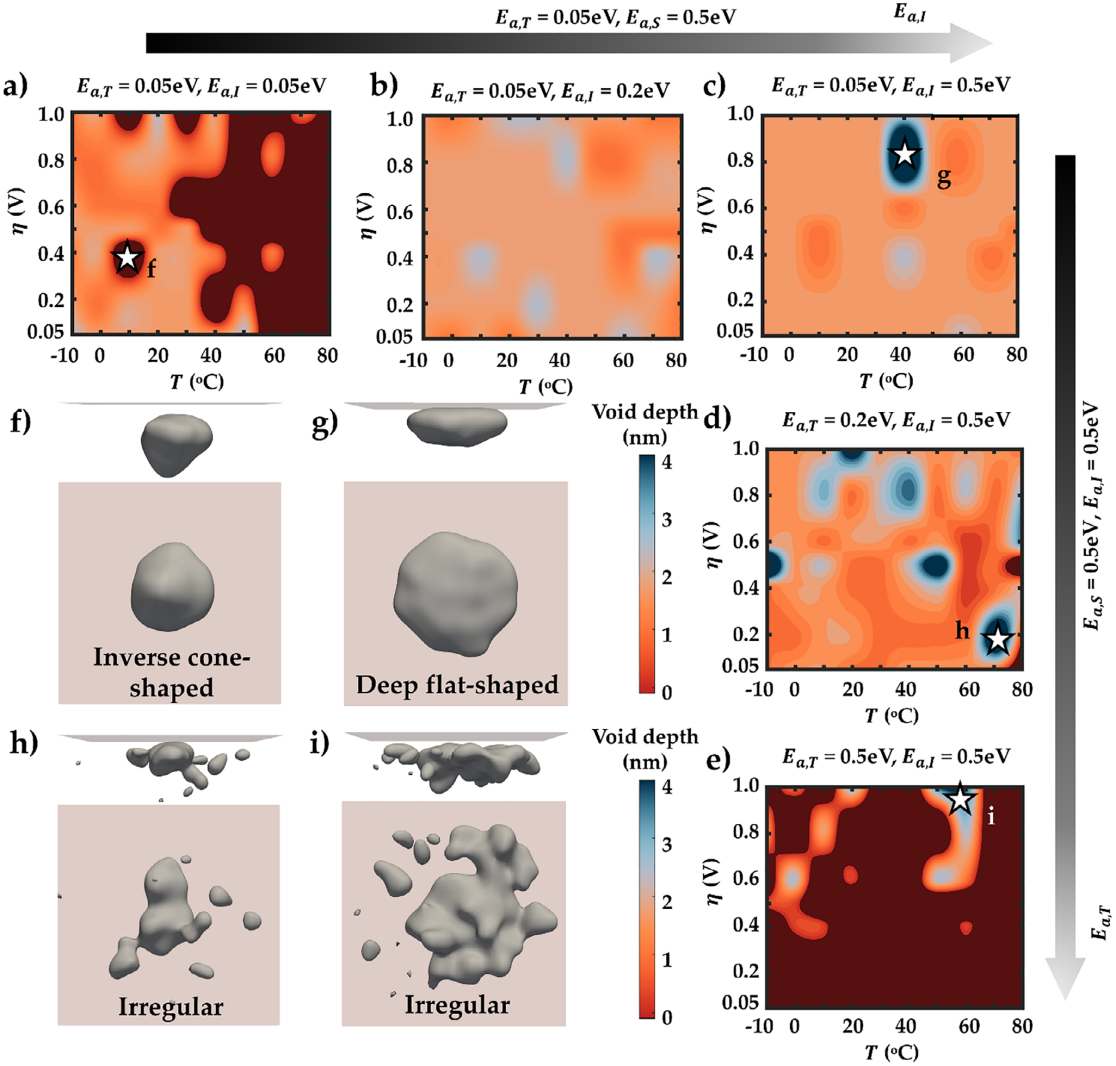
Phase maps of void depth across η and *T*, all at *E*_*a*,*S*_  =  0.5 eV. (a–c) Fixed *E*_*a*,*T*_  =  0.05 eV, with *E*_*a*,*I*_  =  0.05, 0.2, 0.5 eV and (d, e) fixed *E*_*a*,*I*_  =  0.5 eV with *E*_*a*,*T*_  =  0.2, 0.5 eV. (f–i) Distinct single‐void morphologies influenced by various operating temperatures and overpotential values.

The explored temperature range of −10°C to 80°C covers realistic SSB operating conditions, from subzero starts to sustained elevated‐temperature operation. Temperature‐ and pressure‐dependent stripping experiments on Li‐LLZO cells also fall within this range [36, 53]. The predicted reduction in void growth at elevated temperature results from the Arrhenius acceleration of surface diffusion and bulk vacancy diffusion in Equation (8) and therefore represents a transport‐driven healing mechanism. LLZO was chosen as the model electrolyte partly because its interface with Li is kinetically passivated and shows limited interphase growth. In practical cells, the optimal temperature therefore depends on the balance between morphological healing and chemical degradation and is expected to vary with electrolyte chemistry. For garnet‐type oxides, the transport benefit of elevated temperature is more directly captured by the present model. In contrast, sulfide electrolytes can undergo interfacial reduction and form interphases that grow faster at elevated temperature. In some cases, these interphases are mixed conducting and can continue to grow [30, 61, 62], so chemical degradation may offset the morphological benefit of faster transport. Within the present framework, differences between solid electrolyte families and the effects of interphase layers formed under specific temperature and pressure conditions can be represented through changes in the exchange current density, ion transport activation barrier, interface stability, and local current focusing. These effects could be included through a spatially varying reaction rate and are left for future work.

Keeping *E*_*a*,*S*_  =  0.5 eV constant, *E*_*a*,*I*_ is changed from 0.05 to 0.5 eV in Figure 3a–c and the detached void regime reduces significantly. As explained earlier, as the interlayer diffusion dominance reduces, the tendency to form detached voids decreases. An example of the detached void in Figure 3f corresponds to *T*  =  10°C, η  =  0.4 V. However, at *E*_*a*,*I*_  =  0.5 eV, *T*  =  40°C, η  =  0.8 V, there is a single‐void presented in Figure 3g. Next, when *E*_*a*,*T*_ is increased from 0.05 to 0.5 eV, the detached void regime increases as well due to the relative dominance of interlayer diffusion. As terrace diffusion becomes less significant, the shape of the void becomes more irregular and ramified in Figure 3h, i.

### 3.4 Electrochemical Damköhler Number vs. Surface Diffusion on Void Depth

Previously, we examined the impact of the activation barriers associated with surface diffusion modes and of reaction kinetics by varying the overpotential independently. However, it is more practical to study the effects of both simultaneously. Given a constant rate of bulk vacancy diffusion (*E*_*a*,*D*_  =  0.41 eV), the overpotential (η) is varied from 0.05 to 1 V, and the surface diffusion activation barriers (*E*_*a*,*T*_, *E*_*a*,*S*_, *E*_*a*,*I*_) are varied from 0.05 to 0.5 eV. The electrochemical Damköhler number is introduced and defined as below:

(14) $$Da=\frac{k_{0}\;}{k_{D}}\;$$

where, *k*_0_ is the reaction rate, defined in Equation (4), and is dependent on η. And *k_D_* is the bulk vacancy diffusion rate. The *Da* number compares vacancy generation at the interface with vacancy removal into the bulk, and it is independent of the surface diffusion ratio. Since it is independent of the surface diffusion ratio, the two axes in Figure 4 represent two different transport balances. For *Da* ≪ 1, vacancy removal is faster than generation, so vacancies formed near the void edge diffuse into the electrode, and the void shrinks or disappears, leading to the no‐void regime. To clarify, here the *Da*≪1 regime is achieved by lowering the reaction rate, keeping the *k_D_* constant. For *Da* ≫ 1, vacancy generation is faster than removal, causing vacancies to accumulate around the void perimeter. In this regime, the surface diffusion ratio determines the resulting void morphology. Strong terrace diffusion (high *k_T_*/*k_D_*) promotes redistribution along the void footprint and produces compact but irregular voids. Strong step diffusion (high *k_S_*/*k_D_*) promotes vacancy accumulation near the perimeter and produces deeper attached voids. Strong interlayer diffusion (high *k_I_*/*k_D_*) promotes vacancy transport in the depth direction, leading to void detachment at moderate *Da* and complete dissolution when bulk diffusion becomes sufficiently strong.

**FIGURE 4:**
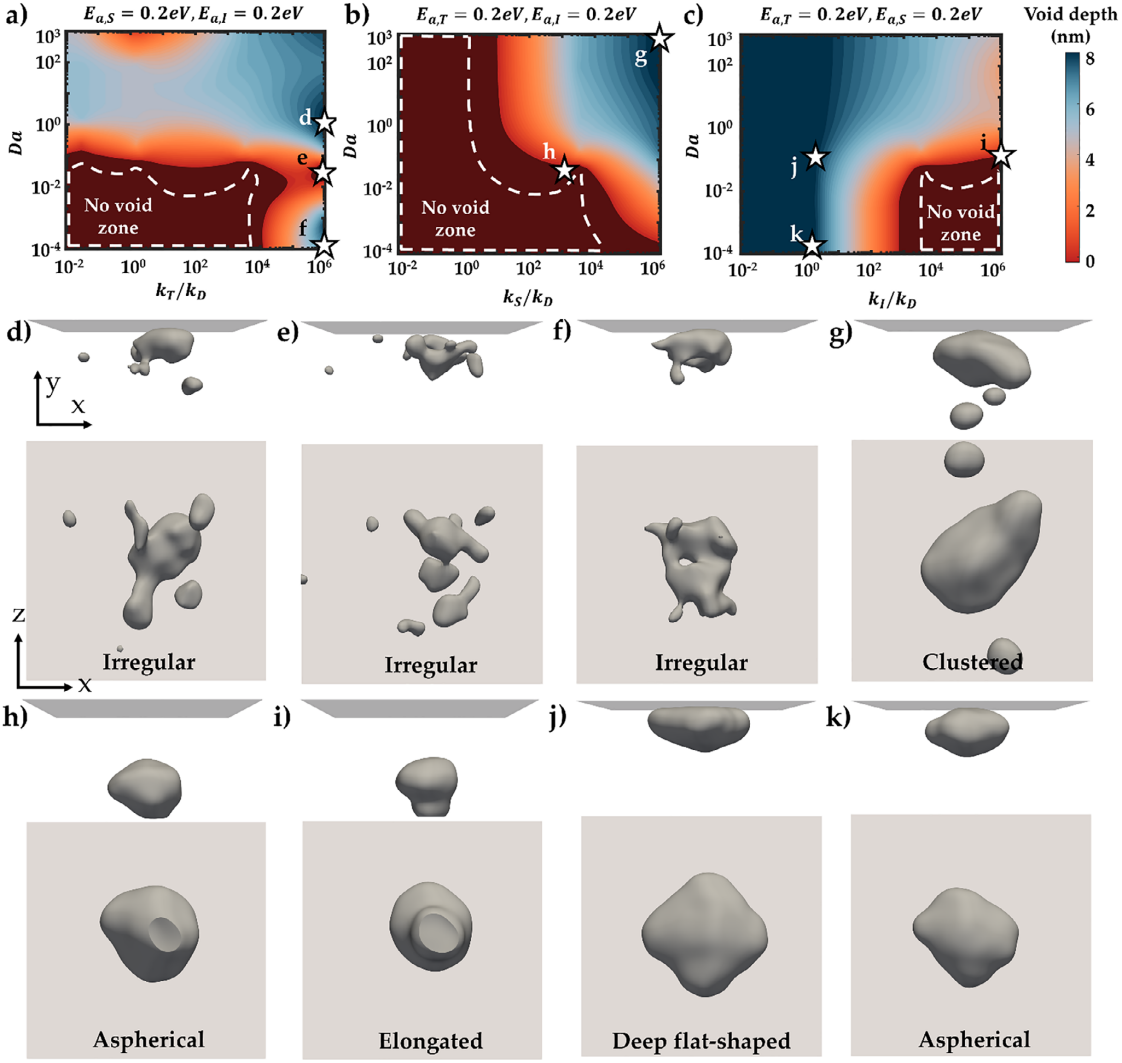
Void depth phase maps as a function of electrochemical Damköhler number (*Da*) and the ratio of surface diffusion rate to bulk vacancy diffusion rate for (a) terrace, (b) step, (c) interlayer diffusion, respectively, where the other activation barriers remain at 0.2 eV. (d)‐(k) Single‐void morphologies near extreme points across the phase plots with varied operating temperature and overpotential.

In Figure 4a–c, the void depth is plotted against two‐dimensional numbers, i.e., *Da* and the ratio *k_SD_*/*k_D_*, where *k_SD_* is the generic surface diffusion rate and in the mode‐specific panels (a–c) it is the terrace, step or interlayer rate (*k_T_*,*k_S_*,*k_I_*), so the horizontal axis reads *k_T_*/*k_D_*, *k_S_*/*k_D_*, and *k_I_*/*k_D_*, respectively. Similarly, as before, the *k_D_* value is kept constant, and *k_SD_* is varied in the mentioned range. Here, the operating temperature is kept constant at room temperature, *T*_0_  =  27°C, the bulk diffusion rate *k_D_* is kept constant, associated with the activation barrier *E*_*a*,*D*_ = 0.41 eV. Given the range of the variables, the value of *Da* changes from 10^−4^ to 10^3^, corresponding to η  =  0.05 V and η  =  1 V respectively and the *k_SD_*/*k_D_* values vary from 10^−2^ to 10^6^, corresponding to *E*_*a*,*SD* _= 0.5 to 0.05 eV. All of the phase plots are segmented into two regions, attached and detached voids. In the dark brown regime, there is another regime (white dotted) with a “no void” regime, which is the same as in Figure 2 and is drawn from the void size phase maps. In all the phase plots, the no‐void regime is towards the *Da*≪1 regime, where the bulk diffusion is dominant, and all the vacancies that constitute the void diffuse towards the bulk and vanish. However, in Figure 4b, the “no void” regime prevails for *k_S_*/*k_D_* < 1 for the given *Da* range, which means that even if the reaction rate is very high, the void is dissolved. A possible explanation may be that interlayer diffusion is more dominant here. The interlayer diffusion rate, *k_I_*/ *k_D_* =  3.3*10^3^ (corresponding to *E*_*a*,*I*_  =  0.2 eV), which falls inside the “no void range” in Figure 4c. This analysis implies that bulk diffusion and interlayer diffusion are two distinct mechanisms that can facilitate void diffusion and cause it to vanish inside the bulk. In Figure 4a,b, the “no void” zone is where *k_SD_*/*k_D_* is smaller (left side of the phase plots), where interlayer diffusion is dominant, and in Figure 4c, the zone is on the right side of the plot for the same reason.

In Figure 4a, at the highest terrace diffusion (*k_T_*/*k_D_* ∼ 10^6^) case, the voids are irregular in shape (Figure 4d–f) as the Li atoms are moving on the free surface. As we move into the higher Da regime, multiple void fragments formed due to more frequent dissolution events. However, in Figure 4e, the void is detached from the interface, as the relative impact of interlayer diffusion becomes more pronounced compared to the other two cases. In Figure 4b, there is a clear transition from the detached void regime to the deepest void regime from low Da, low *k_S_*/*k_D_* to high Da, high *k_S_*/*k_D_* regime. In the top‐right corner of the phase plot, the void is deepest (Figure 4g) because high step diffusion helps void coalescence, and the void volume increases due to a high dissolution rate. However, with moderate surface diffusion and reaction rates, the void travels significantly across the interface. Still, its shape is more spherical due to the combined effect of terrace and step diffusion. In Figure 4c, the regime *k_I_*/*k_D_* < 1 shows a trend in deep voids across the full range of Da, where terrace and step diffusion dominate over interlayer diffusion. In Figure 4j,k, the void is deeper and attached to the interface. In the higher Da case, the void is more spherical because more vacancies are generated at the existing void edge, these vacancies diffuse along the void surface via interlayer diffusion, resulting in a more spherical structure. However, if interlayer diffusion increases at the same Da, the void diffuses as a whole from the interface to the bulk, forming an elongated void. The distinct roles of surface and bulk transport can also be seen from their limiting behaviors. When bulk diffusion dominates, the vacancy cluster spreads into the electrode, and the void gradually disappears. In contrast, the characteristic lateral growth and subsequent deepening of the void are observed only when surface diffusion becomes significant.

### 3.5 Void Clustering and Transition to Percolated Void Structure

The morphological evolution of a single void involves the nucleation, growth, and coalescence of multiple void clusters as the stripping progresses. Within the lattice description, a single vacancy site represents an isolated Li vacancy, a point defect that migrates through bulk diffusion, whereas a void is defined as a cluster of two or more vacancies residing within their 1NN positions (8 1NN sites in a BCC crystal). The cluster volumes are reported as the number of vacancy sites multiplied by the atomic volume of BCC Li, Ω_*Li*_ = *a* ^3^ /2 ≈ 0.0214*nm* ^3^. The void interior is treated as empty space generated by Li depletion, with no gaseous or interphase species present. In Figure 5a–d, the number of void clusters present is calculated and plotted against the corresponding void cluster volume for four instances during stripping, and the corresponding void morphologies are shown in Figure 5e–h. At the start, there is only one void present inside the domain with a volume of 6 nm^3^. Just after stripping starts, multiple void‐nucleation events occur randomly at the interface and along the void edges, as these regions are prone to electro‐dissolution. These newly generated vacancy clusters, predominantly single‐site point defects, are on the order of 10^3^ in number, and the volume of the large void does not change (Figure 5b). The single‐site clusters populate the smallest‐volume bin and act as the nuclei from which voids grow through aggregation and coalescence. In Figure 5c, the newly nucleated voids continue to maintain their individual presence. Meanwhile, multiple voids coalesce, increasing the number of medium‐volume voids, and the large void continues to grow to 7 nm^3^. In this stage, the voids coalesce to form a percolating void structure within the domain. Near the end of the stripping, most of the voids coalesce into the largest central void, increasing its volume to 9 nm^3^. The remaining smaller voids also continue to merge, as reflected in the decrease in their population in Figure 5d. The final void morphology in Figure 5h shows that the void network has retracted from the percolated structure. The coalescence of neighboring clusters is driven by two coupled effects. Current focusing is strongest at the narrow Li ligaments separating adjacent voids, so these bridges undergo preferential dissolution and clusters merge. Curvature‐mediated surface diffusion then transports atoms away from the high‐curvature necks of the merged structure, lowering the total surface energy and consolidating the combined cluster into a compact void. The complete nucleation, growth, and coalescence sequence is shown in Video S1.

**FIGURE 5:**
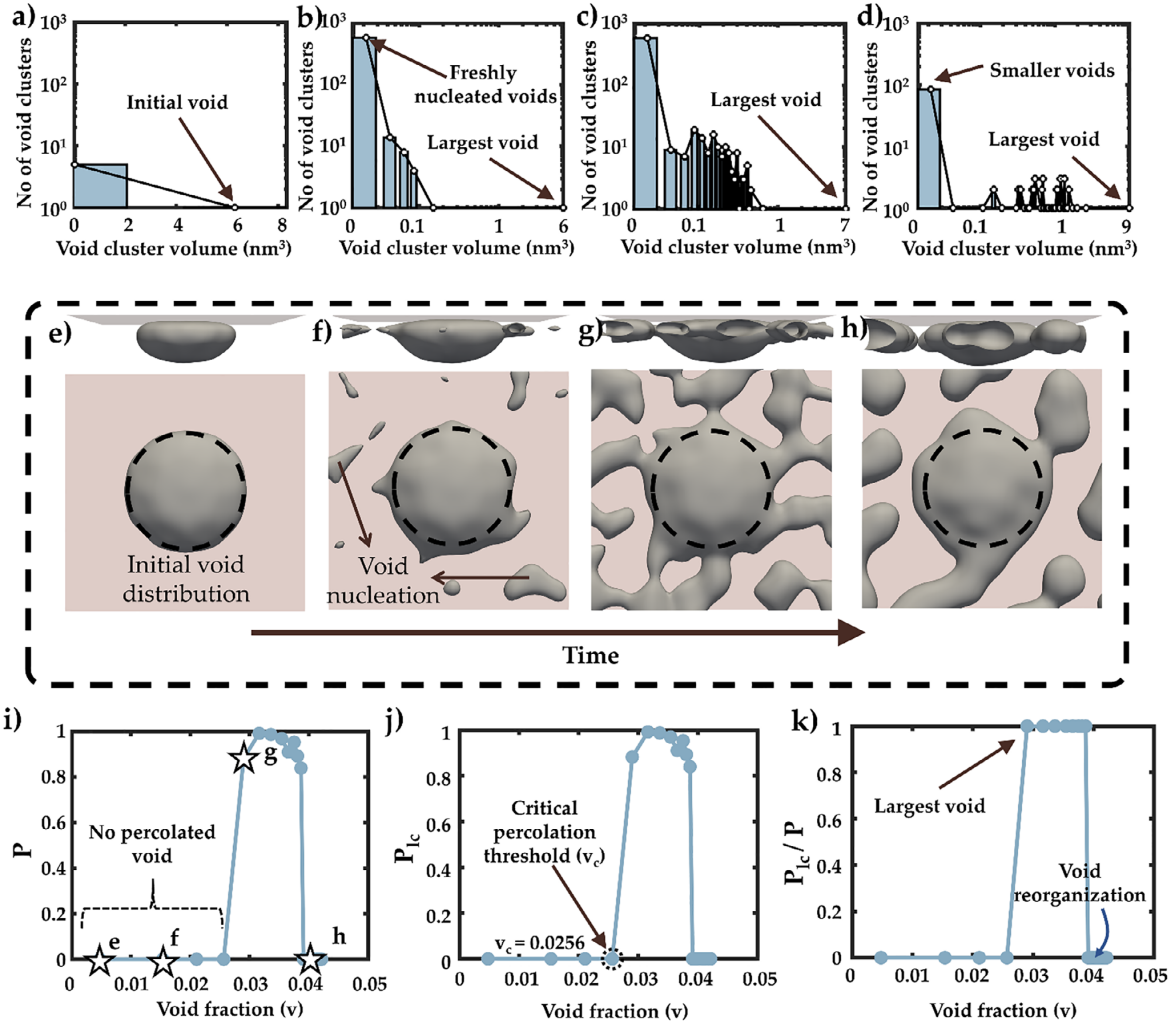
(a–d) Dynamic evolution of cluster density as a function of void cluster volume. (e–h) Side‐view and cross‐sectional view snapshots of the evolution from a single‐void to a percolated network. Percolation order parameter of (i) all clusters, (j) largest cluster, (k) largest cluster fraction as a function of void fraction.

From the earlier results, it is evident that there is a clear transition from a single isolated void to a percolated void structure. To quantify this transition, we introduce two parameters: percolation order parameter (P), and percolation order parameter for the largest cluster (P_lc_), defined as follows:

(15) $$P=\frac{Number\;of\;sites\;in\;all\;percolated\;void\;structures}{Total\;number\;of\;vacancy\;sites}\;$$

(16) $$P_{lc}=\frac{Number\;of\;sites\;in\;the\;largest\;percolated\;void\;structure}{Total\;number\;of\;vacancy\;sites}\;$$

Here *P* represents the fraction of vacancy sites that belong to percolated void structures in a given instance, serving as an order‐parameter‐like measure of the extent of percolation. In Figure 5i, we plotted the evolution of *P* as a function of the void fraction defined earlier. We observed a sudden transition from a zero‐percolation structure to a percolated structure. This transition is characterized in the simulation domain when the void is connected across the periodic boundaries (in either the x or z direction), thereby creating a continuous percolated void structure. This percolating void transition reflects the role of surface diffusion in enabling lateral connectivity of voids across the domain. In Figure 5j, only the largest cluster's percolation order parameter is presented, and the critical void fraction where the transition happens is noted as *v_c_*  =  0.0256. In Figure 5k, the contribution of the largest cluster to the percolated structure (*P_lc_*/*P*, evaluated only when *P*  >  0) is shown, which is 1 throughout the percolation regime, indicating that the percolated void structure consists entirely of a single cluster, consistent with the single‐void morphology at the start of the simulation. The electrochemical consequence of this transition is quantified in Section S4, where the interfacial constriction resistance is estimated directly from the simulated contact morphology using a flux tube formulation. The normalized resistance increases by approximately two orders of magnitude across the percolation window and reaches a maximum at the minimum contact fraction. It then decreases as void detachment restores interfacial contact (Figure S5). This result shows that the geometric percolation transition is accompanied by a measurable increase in interfacial resistance.

### 3.6 Impact of Surface Diffusion Modes on Percolation Order Parameter

In Figure 6, the critical percolation order parameter is calculated over the parameter space, 0.05 eV ≤ *E*_*a*,*T*_, *E*_*a*,*S*_, *E*_*a*,*I*_ ≤ 0.5 eV and the change in the morphologies is shown over the parametric space. Figure 6a–c plots the critical percolation void fraction against the surface diffusion barriers. The black regime in all three phase plots indicates that no percolated void has formed. The complete regimes for *E*_*a*,*T*_  =  0.05, 0.2 eV do not have any percolated void present, which clearly explains that with a high terrace diffusion rate, there is no formation of percolated void. This is because terrace diffusion, coupled with step diffusion, creates few void structures with larger volume, as shown in Figure 6d–f. As the terrace diffusion becomes less dominant, the lateral growth of the void becomes more visible, such as in Figure 6f. In Figure 6c, there is no percolated void regime for low *E*_*a*,*I*_ regime, i.e., where the interlayer diffusion is the strongest. This is because high interlayer diffusion transports the void in the depth direction, increasing its depth and eventually detaching it from the interface. However, *E*_*a*,*T*_  =  0.5 eV, *E*_*a*,*I*_ > 0.2 eV regime provides a significant distribution of percolated voids. A higher critical percolation threshold means that the single‐void structure transitioned to a percolated void at a higher void fraction, which implies that the voids are deeper (Figure 6g,h), compared to when the critical percolation threshold is smaller (Figure 6i). From Figure 6c, a higher percolation threshold is observed at low *E*_*a*,*S*_ values, where deeper voids are formed, and then they transition to a percolated structure. This behavior supports the conclusion that deeper voids form in high‐step‐diffusion regimes. However, a maximum critical percolation void fraction is achieved when interlayer diffusion and step diffusion are combined, as shown in Figure 6g.

**FIGURE 6:**
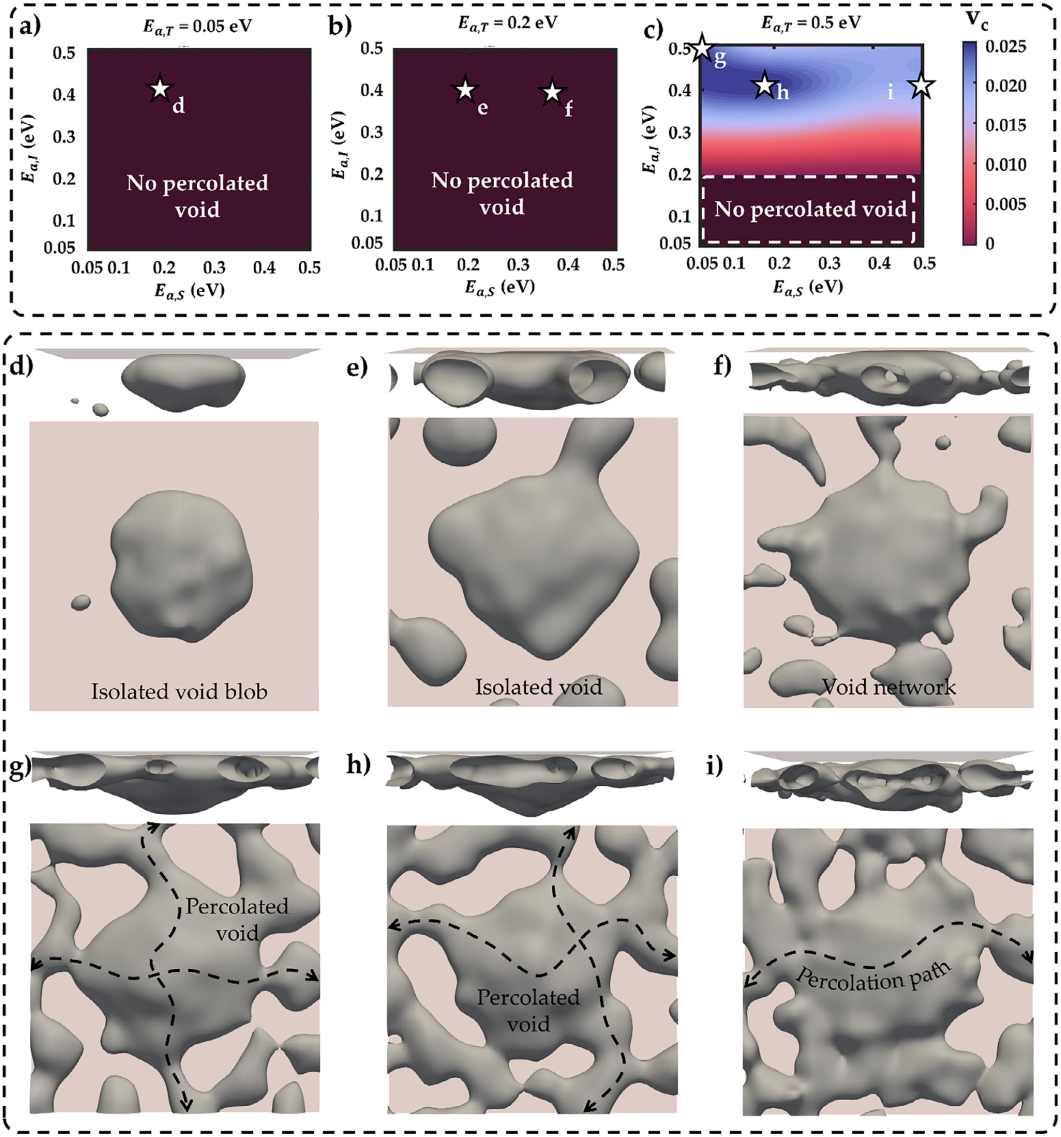
(a–c) Critical percolation order parameter phase map as a function of *E*_*a*,*S*_ and *E*_*a*,*I*_ for *E*_*a*,*T*_  =  0.05, 0.2, 0.5 eV. Distinct void morphologies with (d–f) no percolation and (g–i) percolation.

The simulated void dynamics are qualitatively consistent with observations from operando and in situ experiments. The model predicts that the void first expands laterally at nearly constant depth and then deepens through vacancy redistribution. This behavior is consistent with the pore evolution inferred from operando dilatometry, where pores form during stripping, spread along the interface, and then grow deeper [34]. The threshold dissolution rate separating the healing and void‐growth regimes in the Damköhler analysis is also consistent with the critical stripping current above which voids begin to accumulate [31, 32]. In addition, the predicted dependence of void morphology on the dissolution rate agrees with trends observed by in‐situ microscopy [35]. With continued stripping, vacancy clusters nucleate, grow, and coalesce to form a connected void network, leading to a strong decrease in contact area. This trend is consistent with the progressive void accumulation reported experimentally, which reduces the active contact area and increases the local current density at the remaining contact points [31]. It also agrees with operando tomography results showing that stripping‐induced contact loss is a major source of current constriction and cell failure, rather than interphase growth [33]. The formation of detached subsurface voids and the partial healing of attached voids under enhanced surface diffusion are also consistent with the partial recovery of dewetted interfaces observed after thermal treatment [36]. These comparisons are necessarily qualitative because the present model focuses on the early‐stage evolution of a single nanoscale void, while imaging experiments generally capture micrometer‐scale voids at later stages of degradation. The percolation transition is also expected to depend on stack pressure. Creep‐assisted Li transport can reduce the number of vacancies retained near the interface and therefore limit the lateral connection between neighboring voids. Increasing stack pressure may thus shift the onset of percolation to higher void fractions and, at sufficiently high pressure, may suppress the formation of a percolated void network.

## 4 Conclusion

In this work, a mesoscale modeling framework is established to resolve the dynamical evolution of individual void morphology at the Li metal anode‐SE interface. The interplay among bulk vacancy diffusion, reaction kinetics at electrochemically active sites, and the distinct surface diffusion modes dictates the shape, size, and final morphology of a single void. Through this study we have established that an isolated void first grows laterally along the interface under strong current constriction and subsequently deepens through vacancy redistribution, with the overall growth governed by intricately coupled contributions from the surface diffusion modes. Terrace and step diffusion form compact void clusters along the interface, whereas interlayer diffusion directs transport into the depth rather than along the interface and therefore tends to produce voids that detach from the interface, enabling contact recovery. The competing effects of temperature and overpotential also shape the morphology, where stronger interlayer diffusion promotes detached voids while reduced interlayer dominance and weaker terrace diffusion drive the void toward attached, irregular structures. Prolonged stripping raises the vacancy flux within the electrode and increases the void fraction, with strong interlayer diffusion yielding highly fragmented clusters and strong step diffusion yielding large coalesced clusters. Over time, the single void transitions to a percolated structure at a critical void fraction of *v_c_*  =  0.0256 for baseline surface diffusion rates. Because terrace and step diffusion preserve compact, isolated clusters rather than a connected network, a single‐void morphology dominates over a percolated one. Strong terrace diffusion compacts the void into an isolated structure, whereas strong interlayer diffusion drives it into the depth and detaches it from the interface. A percolated network therefore emerges only when both modes are sufficiently weak, that is, for *E*_*a*,*T*_  =  0.5 eV, *E*_*a*,*I*_ > 0.2 eV, where voids are retained near the interface and link laterally. Within this regime, step diffusion remains the active and controlling mechanism that dictates the lateral connectivity of the percolated network, where strong step diffusion delays the onset of the percolated structure. Therefore, based on the dominance of the surface diffusion modes, the percolated void morphology trend changes significantly. Thus this work provides the theoretical baseline for the optimum surface diffusion modes that dictate void morphology, thereby informing design strategies for highly efficient SSBs. Mechanical effects, including stack pressure and Li creep, are not included in the present model. Li creep is expected to supply material toward the interface, which can reduce vacancy accumulation and slow void growth. Increasing stack pressure may therefore enhance void detachment by facilitating plastic deformation of Li metal. The present results represent the pressure‐free limit and therefore provide a conservative bound for void growth. In the KMC framework, these effects could be included through stress‐dependent hopping barriers or an additional creep‐driven atomic flux toward the interface. Incorporating this mechanical coupling is an important direction for future work.

## Author Contributions

Conceptualization, S. B., G. L., and P. P. M.; Methodology, S. B.; Formal Analysis, S. B.; Visualization, S. B.; Writing – original draft, S. B.; Writing – review and editing, S. B., B. S. V., G. L., and P. P. M.; Supervision, P. P. M.

## Conflicts of Interest

The authors declare no conflicts of interest.

## Supporting information




**Supporting File 1**: advs78028‐sup‐0001‐SuppMat.docx.

**Supporting File 2**: advs78028‐sup‐0002‐VideoS1.mp4.

## Data Availability

All data in this manuscript is available upon request from the lead contact.
