# Supplementary material for: Mechanistic Insights Into Instability From Single Void Dynamics at the Lithium Metal‐Solid Electrolyte Interface
Source: Adv Sci (Weinh). 2026 Sep 30:e78028. Online ahead of print. doi: 10.1002/advs.78028 (PMC13626669; doi:10.1002/advs.78028)
Supplement: Supplementary file 1 [file ADVS-9999-e78028-s002.docx]

**Supporting Information**

**Mechanistic Insights into Instability from Single Void Dynamics at the Lithium Metal-Solid Electrolyte Interface**

Sourim Banerjee^1^, Bairav S. Vishnugopi^1^, Guang Lin^1,2^, Partha P. Mukherjee^1,*^

^1^ School of Mechanical Engineering, Purdue University, West Lafayette, IN 47907, United States

^2^ Department of Mathematics, Purdue University, West Lafayette, IN 47907, United States

**Correspondence:* pmukherjee@purdue.edu

**S1. Mesoscale coarse-grained framework**

A coarse-grained mesoscale framework is constructed to follow the time-dependent behavior of the electrochemical system. Within this Kinetic Monte Carlo (KMC) scheme, four governing processes are represented, i.e., ion transport through the solid electrolyte (SE), oxidation of metal atoms at the SE-lithium (Li) metal boundary, vacancy migration from the boundary into the electrode bulk, and surface diffusion of Li atoms. A rate constant for each process is evaluated at every lattice site across the domain. These four site-level rate constants are then aggregated into the corresponding total rate constants described below.

For metal ion transport in the electrolyte,

$k_{1}=\sum_{i=1}^{N_{1}} k_{IT}^{i}$	(S1)

Here, $N_{1}$ denotes the number of metal ions residing in the electrolyte domain, and $k_{1}$ is the total ion transport rate obtained accordingly.

For the oxidation of Li metal atoms,

$k_{2}=\sum_{i=1}^{N_{2}} k_{R}^{i}$	(S2)

Here, $N_{2}$ corresponds to the metal atoms at the electrode-electrolyte boundary, yielding the total electrochemical reaction rate $k_{2}$ . For vacancy migration through the bulk,

$k_{3}=\sum_{i=1}^{N_{3}} k_{D}^{i}$	(S3)

Here, $N_{3}$ gives the number of vacancies in the domain and the total bulk diffusion rate $k_{3}$ is calculated. For the surface diffusion of Li atoms at the Li metal top layer,

$k_{4}=\sum_{i=1}^{N_{4}} k_{SD}^{i}$	(S4)

Here, $N_{4}$ gives the number of Li surface atoms undergoing terrace, step, or interlayer diffusion. Combining the four contributions, $k_{1}$, $k_{2}$, $k_{3}$ and $k_{4}$, the overall rate constant is assembled as,

$k_{tot}=\sum_{i=1}^{4} k_{i}$	(S5)

A random number $r_{i}$ drawn uniformly between 0 to 1 is scaled by the total rate $k_{tot}$. The event list is then traversed, and the first event whose cumulative rate exceeds this scaled value (${r_{1}k}_{tot}$) is selected and the system advances according to that event.

The duration of the chosen event is set using a second random number $r_{2}$,

$\delta t=-\frac{1}{k_{tot}}ln(r_{2})$	(S6)

The algorithmic sequence governing the system’s temporal evolution is depicted in the flowchart of Figure S1.

Figure S1. Flowchart of the KMC algorithm used to advance the electrochemical system in time.

Table S1 summarizes the parameters used in the coarse-grained KMC model. The surface diffusion activation barriers ($E_{a,T}, E_{a,S}, E_{a,I}$) are based on first-principles calculations for BCC Li surfaces. A detailed mechanistic description of the terrace, step, and interlayer diffusion modes, together with schematic illustrations of the corresponding pathways, is provided in the earlier work by Banerjee et al. ^[1]^. Since first-principles activation barriers can vary with crystallographic facet and step orientation, the parametric sweeps in the present study vary these barriers from 0.05 to 0.50 eV to account for this variability.

Table S1. Model parameters adopted in the KMC simulations.

|  | Parameters | Values | Units |
| --- | --- | --- | --- |
|  | System dimensions | 50 × 50 × 50 lattice  supercell | - |
| $a_{Li}$ | Lattice size of Li | 3.5 | Å |
| $E_{a,D}$ | Vacancy diffusion activation barrier^[2]^ | 0.41 | eV |
| $E_{a,IT}$ | Ion transport activation barrier^[3]^ | 0.35 | eV |
| $E_{a,T}$ | Terrace surface diffusion activation barrier^[4]^ | 0.3 | eV |
| $E_{a,S}$ | Step surface diffusion activation barrier^[4]^ | 0.15 | eV |
| $E_{a,I}$ | Interlayer surface diffusion activation barrier^[4]^ | 0.5 | eV |
| F | Faraday constant | 96485 | C/mol |
| L | Domain dimension | 17.5 | nm |
| $\nu$ | Hopping frequency^[5]^ | 2×10^12^ | 1/s |
| $k_{b}$ | Boltzmann constant | 8.617×10^-5^ | eV/K |
| $D_{Li}$ | Bulk diffusivity of Li metal^[6]^ | 2×10^-14^ | m^2^/s |
| $i_{0}$ | Exchange current density for Li-LLZO interface^[7]^ | 0.5 | mA/cm^2^ |
| $\alpha, \beta$ | Charge transfer coefficients | 0.5 | - |

**S2. Sensitivity analysis of the surface diffusion barriers on void depth**

A global sensitivity analysis was performed using the variance-based Sobol^[8]^ method implemented in SALib.^[9]^ The three surface diffusion activation barriers-$E_{a,T}$ (terrace), $E_{a,S}$ (step), $E_{a,I}$ (interlayer) were each sampled uniformly over [0.05, 0.5] eV, while the bulk vacancy diffusion barrier ($E_{a,D}=0.41$ eV), and applied overpotential ($\eta=0.8 V$) were held fixed. Input samples were generated using the Saltelli quasi-random scheme with scrambling enabled (N=2048, D (number of parameters) = 3), yielding a total of N×(2D+2) = 16384 KMC simulations. Each simulation was run independently, and the scalar model output used for index estimation was the final void depth $b$ at the end of the stripping process. Sobol indices were estimated using SALib’s Saltelli estimator with bootstrap confidence intervals reported as error bars in Figure S2(a). The Sobol indices, i.e., first-order $\left( S_{E_{a,i}} \right)$, total order $\left( S_{T_{E_{a,i}}} \right)$ and the second-order $\left( S_{E_{a,i},E_{a,j}} \right)$ indices are defined as follows,

$b=f(E_{a,T}, E_{a,S}, E_{a,I})$	(S7)

$V\left( b \right)=\sum_{i=T,S,I} V_{E_{a,i}}+\sum_{\begin{aligned} i,j=T,S,I \\ i<j \end{aligned}} V_{E_{a,i},E_{a,j}}+V_{T,S,I}$	(S8)

$V_{E_{a,i}}=\mathbb{V}_{E_{a,i}}\left( \mathbb{E[}b|E_{a,i}] \right), i=T,S,I$	(S9)

$V_{E_{a,i},E_{a,j}}=\mathbb{V}_{E_{a,i},E_{a,j}}\left( \mathbb{E}\left[ b | E_{a,i},E_{a,j} \right] \right)-V_{E_{a,i}}-V_{E_{a,j}}$	(S10)

$S_{E_{a,i}}=\frac{V\left( E_{a,i} \right)}{V\left( b \right)}$	(S11)

$S_{E_{a,i}, E_{a,j}}=\frac{V\left( E_{a,i}, E_{a,j} \right)}{V\left( b \right)}$	(S12)

$S_{T_{E_{a,i}}}=1-\frac{V\left( E_{a,\sim i} \right)}{V\left( b \right)}$	(S13)

where, $\mathbb{V}_{E_{a,i}}(\cdot)$ denotes the variance operator taken over $E_{a,i}$, $\mathbb{E[}b|E_{a,i}]$ denotes the conditional expectation of $b$ given $E_{a,i}$ averaged over all other inputs, $V(b)$ is the total unconditional variance of $b$, and $E_{a,\sim i}$ represents all the activation barriers except the $i$^th^ activation barrier. In Figure S2(a), the first and the total Sobol indices are plotted for all three distinct surface diffusion mechanisms. All the first-order indices are very small ($S_{E_{a,T}}=0.088, S_{E_{a,I}}=0.074, S_{E_{a,S}}=0.035$), but nonzero, indicating that each activation barrier has a weak yet measurable direct contribution to the variance of final void depth. Their sum ($\sum S_{i}\approx0.20$) indicates that only about 20% of the output variance stems from independent first-order effects, with the remaining ~80% arising from interactions, directly establishing that the system is interaction-dominated. However, the first-order and pairwise contributions do not, by themselves, account for the displayed total-order indices, indicating substantially higher-order coupling among all three pathways. In Figure S2(b), the largest reported pairwise interaction is between terrace and interlayer diffusion ($S_{E_{a,T}, E_{a,I}}$= 0.17), followed by interlayer-step ($S_{E_{a,S}, E_{a,I}}=0.098$) and terrace-step ($S_{E_{a,T}, E_{a,S}}=0.081$) interactions. The residual three-way interaction $S_{E_{a,T},E_{a,S},E_{a,I}}=0.455$, computed as $1-S_{E_{a,T}}-S_{E_{a,S}}-S_{E_{a,I}}-S_{E_{a,T},E_{a,S}}-S_{E_{a,T},E_{a,I}}-S_{E_{a,S},E_{a,I}}$, indicates that nearly half of the total output variance arises from simultaneous coupling among all three diffusion pathways. With the first-order, pairwise, and the three-way contributions summing to unity as required for a complete variance decomposition.

The total-order Sobol indices for terrace and interlayer diffusion are $S_{T, E_{a,T}}=1.05\pm0.43$ and $S_{T, E_{a,I}}=1.22\pm0.44$, respectively, with $S_{T, E_{a,S}}=0.74\pm0.19$ for step diffusion (95% bootstrap confidence intervals). The values for $E_{a,T}$ and $E_{a,I}$ nominally exceed unity, which is a known finite-sample artifact of the Saltelli estimator when applied to stochastic simulation models. In KMC, each run produces a statistically distinct void morphology even under identical input parameters, owing to the inherent randomness of the thermally activated hopping sequence. This run-to-run stochasticity introduces an additional source of variance into the model output $Y=b$ that is indistinguishable from a parameter-driven variance by the Saltelli estimator, causing the estimated total-order contributions to collectively exceed unity. Importantly, even the lower bounds of the 95% confidence intervals for all three parameters remain well above 0.5 (0.62, 0.55, and 0.78 for $E_{a,T}$, $E_{a,S}$, and $E_{a,I}$ respectively), confirming that the qualitative conclusion is robust with respect to estimator uncertainty.

This analysis indicates that single-void depth is primarily governed by the coupling between in-plane (terrace) and out-of-plane (interlayer) diffusion pathways. This system is strongly interaction-dominated, with negligible independent contributions from individual surface diffusion pathways. Terrace and interlayer diffusion emerge as the primary controlling mechanisms, not through their isolated effects but via strong cooperative coupling. The dominant interaction between terrace and interlayer diffusion highlights the inherently three-dimensional nature of vacancy transport governing void evolution, while step diffusion plays a secondary, modulating role.

Figure S2. Sensitivity of void depth to the surface diffusion activation barriers. (a) First-order and total order Sobol indices corresponding to three distinct surface diffusion modes, $E_{a,T}$, $E_{a,S}$, and $E_{a,I}$. (b) Second-order Sobol interaction indices for the surface diffusion activation barriers.

Convergence of the estimated sensitivity indices was verified by recomputing the first-order and total-order indices using nested subsets of the Saltelli sample with (N = 128, 256, 512, 1024, 2048) (Figure S3). The 95% bootstrap confidence intervals become substantially narrower as the sample size increases, providing further evidence of convergence. The estimated indices also stabilize within the corresponding confidence intervals for ($N\geq1024$), confirming that the reported values are sufficiently converged.

The strong terrace-interlayer interaction reflects the competition between lateral transport along the interface and transport in the depth direction, which together influence whether the void remains attached or detaches from the interface. The interlayer-step and terrace-step interactions are weaker and represent secondary coupling between the surface diffusion pathways during void evolution.

Figure S3. Convergence of the Sobol indices with sample size. (a) First-order and (b) total-order indices for the three surface diffusion activation barriers, recomputed on nested subsets of the Saltelli sample with N = 128, 256, 512, 1024, and 2048 base samples. Error bars denote 95% bootstrap confidence intervals.

**S3.** **Sensitivity to the initial void geometry**

Simulations were repeated using hemispherical initial voids with radii of 1.05nm, 1.40nm, 1.75nm, 2.10nm, 2.45nm, 2.80nm, and 3.15nm (Table S2). Additional simulations were performed using semi-ellipsoidal initial voids with aspect ratios ($h/R$) ranging from 0.2 to 8 at a fixed void volume equal to that of the baseline hemisphere with a radius of 1.75 nm (Table S3).

In all cases, the void follows the same general evolution of lateral expansion followed by redistribution-driven deepening and finally remains as an attached, flattened void with ($\psi< 1$) (Figure S4(c), (d)). The final circularity index converges to a narrow range of approximately 0.65 to 0.73 for all initial radii and for ($h/R \geq0.5$) (Figure S4(e), (f)). This indicates that the final footprint compactness is mainly controlled by the diffusion kinetics rather than the initial void geometry. The final depth changes smoothly with the initial radius in the size sweep and with the initial seed depth in the fixed-volume aspect-ratio sweep (Figure S4(a), (b)).

None of the initial conditions shifts the system between the attached, detached, and dissolved regimes under the baseline kinetic conditions, showing that the main conclusions are not specific to the selected initial geometry. Larger initial voids were not considered because, for ($R > 3.15nm$) or ($h/R > 8$), the void approaches the periodic boundaries or the electrode depth of the domain. Under these conditions, the finite simulation cell can no longer accommodate the initial void without introducing boundary effects.

Table S2. Final morphology descriptors for hemispherical initial voids of varying radius.

| Initial radius R (nm) | Final void depth (nm) | Final $\psi$ | Final $\chi$ |
| --- | --- | --- | --- |
| 1.05 | 1.24 | 0.435 | 0.686 |
| 1.40 | 1.36 | 0.372 | 0.704 |
| 1.75 | 1.48 | 0.318 | 0.697 |
| 2.10 | 1.57 | 0.275 | 0.682 |
| 2.45 | 1.75 | 0.267 | 0.673 |
| 2.80 | 1.92 | 0.262 | 0.730 |
| 3.15 | 2.14 | 0.261 | 0.729 |

Table S3. Final morphology descriptors for semi-ellipsoidal initial voids of varying aspect ratio h/R at fixed void volume, equal to that of the baseline hemisphere of radius 1.75 nm (294 lattice sites, ≈ 6.3 nm³).

| Initial aspect ratio (h/R) | Initial radius R (nm) | Initial depth h (nm) | Final void depth (nm) | Final $\psi$ | Final $\chi$ |
| --- | --- | --- | --- | --- | --- |
| 0.2 | 2.99 | 0.60 | 1.05 | 0.185 | 0.220 |
| 0.25 | 2.78 | 0.69 | 1.08 | 0.165 | 0.215 |
| 0.5 | 2.20 | 1.10 | 1.40 | 0.280 | 0.725 |
| 1 | 1.75 | 1.75 | 1.48 | 0.318 | 0.697 |
| 2 | 1.39 | 2.78 | 1.42 | 0.330 | 0.680 |
| 3 | 1.21 | 3.64 | 1.57 | 0.380 | 0.670 |
| 4 | 1.10 | 4.41 | 1.58 | 0.385 | 0.675 |
| 6 | 0.96 | 5.78 | 1.75 | 0.440 | 0.690 |
| 8 | 0.88 | 7.00 | 2.47 | 0.640 | 0.655 |

Figure S4. Sensitivity of the final void morphology to the initial void geometry. (a, c, e) Final void depth, vertical shape factor $\psi$, and circularity index $\chi$ as functions of the initial void radius $R$ for hemispherical seeds with $R$ = 1.05 to 3.15 nm. (b, d, f) The same descriptors as functions of the initial aspect ratio $h/R$ for semi-ellipsoidal seeds with $h/R$ = 0.2 to 8 at a fixed void volume equal to that of the baseline hemisphere with $R$ = 1.75 nm. All initial conditions terminate as attached, flattened voids with $\psi< 1$. The final circularity index converges to approximately 0.65 to 0.73 for all initial radii and for $h/R \geq0.5$, while only the most oblate seeds with $h/R \leq0.25$ retain a less compact footprint, indicating that the final footprint compactness is controlled by the transport kinetics rather than the initial geometry.

**S4. Interfacial constriction resistance from the simulated contact morphology**

During stripping, ionic current crosses the interface only through the remaining Li-LLZO contact area. As the contact area decreases, current lines become concentrated within the remaining contact patches, producing an additional constriction resistance beyond the bulk resistance of the solid electrolyte. To connect the geometric percolation transition to a measurable electrochemical quantity, the constriction resistance is estimated directly from the simulated interface occupancy maps. At each snapshot, the contact fraction $\phi$ is defined as the fraction of interface sites where Li remains in contact with the solid electrolyte. Contact patches are identified on the interface plane using in-plane nearest-neighbor connectivity with periodic boundaries, while the instantaneous void fraction $v$ is evaluated over the same Li domain used for the percolation analysis in the main text.

For an isolated circular contact of radius $a$ on an otherwise insulating plane, the constriction resistance in a medium with ionic conductivity $\kappa$ is given by $R=1/4\kappa a$.^[10]^ A parallel sum over $N$ contact patches gives,

$R_{disk}=\frac{1}{4\kappa\sum a_{i}}$	(S14)

$a_{i}=\left( \frac{A_{i}}{\pi} \right)^{1/2}$	(S15)

where $A_{i}$ is the area of each contact patch. This formulation treats the patches as independent and predicts a decrease in resistance during contact loss because fragmentation of a fixed contact area into many smaller patches increases $\sum a_{i}$. The proximity-corrected formulation of Greenwood adds pairwise interaction terms between the patches and recovers the correct trend, but the resistance increase remains below a factor of two.^[11]^ Both formulations therefore underestimate the current constriction at low contact fractions.

The flux tube formulation avoids this limitation by treating the domain as a single flux tube in which the total contacted area is represented by an equivalent circular contact,

$a_{eff}=\left( \frac{\phi A}{\pi} \right)^{1/2}$	(S16)

Additionally, $\epsilon$ represents the ratio of the equivalent contact radius in the Equation (S16) to the flux tube radius $b=\left( \frac{A}{\pi} \right)^{1/2}$ of the entire domain and therefore,

$\epsilon=\frac{a_{eff}}{b}=\phi^{\frac{1}{2}}$	(S17)

The constriction resistance is then written as

$R_{cond}=\frac{\left( 1-\epsilon\right)^{3/2}}{4\kappa a_{eff}}$	(S18)

where the factor $\left( 1-\epsilon\right)^{3/2}$ accounts for the interaction between the constricted current lines and the flux tube boundary.^[12]^ Equation (S18) approaches the isolated-contact result as $\epsilon\to0$ and vanishes at full contact. Normalizing the resistance by its initial value, $R_{cond}/R_{cond,0}$, removes the dependence on $\kappa$, so the normalized resistance depends only on the simulated contact fraction. Where, $R_{cond,0}$ is the resistance at the beginning of the simulation with the single hemispherical void.

Figure S5(a) shows the evolution of $\phi$ with $v$. Along the contact-loss branch, $\phi$ decreases from approximately 0.97 to a minimum of approximately 0.42. Along the recovery branch, $\phi$ increases to approximately 0.72 as voids redistribute along the interface, even though $v$ continues to increase. Figure S5(b) shows the normalized constriction resistance as a function of $\phi$. The contact-loss and recovery branches collapse onto a single curve, confirming that Equation (S18) gives a single-valued relation between $R_{cond}$ and the contact fraction. Figure S5(c) shows the same resistance as a function of $v$ , where the trajectory forms a loop. The resistance increases by roughly two orders of magnitude across the percolation window, reaching approximately 100 times its initial value at the critical void fraction ($v_{c}=0.0256$). It peaks near 150 times its initial value at the minimum contact fraction and then decreases along the recovery branch.

The apparent hysteresis with $v$ arises from the evolving interfacial morphology, since the same void fraction can correspond to different contact states before and after void redistribution. The percolation threshold and the minimum contact fraction therefore represent two different stages of the evolution. The percolation threshold marks the formation of a laterally connected void network, while the minimum contact fraction marks the point at which contact recovery due to void detachment begins to overcome continued vacancy generation at the interface. The maximum resistance occurs near this minimum in contact area.

The single flux tube approximation is expected to be more accurate at moderate contact fractions and more qualitative near full contact. However, the main trend remains unchanged, with a relatively small resistance change at high contact fractions followed by a sharp increase across the percolation window. This strong increase in constriction resistance provides an electrochemical signature of the geometric transition and is consistent with the growing polarization observed experimentally as interfacial contact is lost during stripping.^[13]^

Figure S5. Contact resistance evolution with void percolation behavior. (a) Contact fraction $\phi$ as a function of void fraction $v$, showing the contact-loss branch with filled circles and the detachment-driven recovery branch with open circles. (b) Normalized constriction resistance $R_{cond}/R_{cond,0}$, calculated using Equation (S18), as a function of $\phi$. The loss and recovery branches collapse onto a single curve. (c) $R_{cond}/R_{cond,0}$ as a function of $v$, reaching approximately 100 times its initial value at ($v_{c}=0.0256$) and peaking near the minimum contact fraction.

Video S1 (included as separate attachment). Nucleation, growth, and coalescence of vacancy clusters during stripping at $E_{a,D}=0.41eV, E_{a,T}=0.3eV,E_{a,S}=0.15eV,E_{a,I}=0.5eV, \eta=0.8V, T=27ᵒC$, showing preferential dissolution of the Li ligaments between neighboring clusters and their consolidation into the percolated structure. Playback spans dimensionless time t̄ = 0 to 3.6 (101 frames).

**References**

[1]	S. Banerjee, B. S. Vishnugopi, P. P. Mukherjee, *Advanced Science* **2026**, *13*. <https://doi.org/10.1002/advs.202515827>.

[2]	M. Jäckle, A. Groß, *J. Chem. Phys.* **2014**, *141*. <https://doi.org/10.1063/1.4901055>.

[3]	B. Andriyevsky, K. Doll, T. Jacob, *Mater. Chem. Phys.* **2017**, *185*, 210–217. <https://doi.org/10.1016/j.matchemphys.2016.10.025>.

[4]	M. Jäckle, K. Helmbrecht, M. Smits, D. Stottmeister, A. Gross, *Energy & Environmental Science* **2018**, *11*, 3400–3407. <https://doi.org/10.1039/c8ee01448e>.

[5]	O. Borodin, G. V. Zhuang, P. N. Ross, K. Xu, *Journal of Physical Chemistry C* **2013**, *117*, 7433–7444. <https://doi.org/10.1021/jp4000494>.

[6]	E. Dologlou, *Glass Phys. Chem* **2010**, *36*, 570–574. <https://doi.org/10.1134/S1087659610050056>.

[7]	T. Krauskopf, H. Hartmann, W. G. Zeier, J. Janek, *Acs Applied Materials & Interfaces* **2019**, *11*, 14463–14477. <https://doi.org/10.1021/acsami.9b02537>.

[8]	I. M. Sobol, *Mathematics and Computers in Simulation* **2001**, *55*, 271–280. <https://doi.org/Doi> 10.1016/S0378-4754(00)00270-6.

[9]	a)J. Herman, W. Usher, *The Journal of Open Source Software* **2017**, *2*. <https://doi.org/10.21105/joss.00097>; b)T. Iwanaga, W. Usher, J. Herman, *Socio-Environmental Systems Modelling* **2022**, *4*. <https://doi.org/10.18174/sesmo.18155>.

[10]	J. S. Newman, K. E. Thomas-Alyea, *Electrochemical systems*, 3rd ed., J. Wiley, Hoboken, N.J, **2004**.

[11]	J. A. Greenwood, *British Journal of Applied Physics* **1966**, *17*, 1621–1632. [https://doi.org/10.1088/0508-3443/17/12/310](https://doi.org/https://doi.org/10.1088/0508-3443/17/12/310).

[12]	M. G. Cooper, B. B. Mikic, Yovanovi.Mm, *Int. J. Heat Mass Transfer* **1969**, *12*, 279–&. <https://doi.org/Doi> 10.1016/0017-9310(69)90011-8.

[13]	J. A. Lewis, F. J. Q. Cortes, Y. Liu, J. C. Miers, A. Verma, B. S. Vishnugopi, J. Tippens, D. Prakash, T. S. Marchese, S. Y. Han, C. Lee, P. P. Shetty, H. W. Lee, P. Shevchenko, F. De Carlo, C. Saldana, P. P. Mukherjee, M. T. McDowell, *Nature Materials* **2021**, *20*, 503–510. <https://doi.org/10.1038/s41563-020-00903-2>.
